# Quantifying greenhouse gas emissions from global aquaculture

**DOI:** 10.1038/s41598-020-68231-8

**Published:** 2020-07-15

**Authors:** Michael J. MacLeod, Mohammad R. Hasan, David H. F. Robb, Mohammad Mamun-Ur-Rashid

**Affiliations:** 1 Scotland’s Rural College, Edinburgh, UK; 2 Aquaculture Branch, FAO Fisheries and Aquaculture Department, Rome, Italy; 3 Cargill Animal Nutrition and Health, Aquaculture Business, Surrey, UK; 4 WorldFish, Dhaka, Bangladesh

**Keywords:** Climate sciences, Environmental sciences

## Abstract

Global aquaculture makes an important contribution to food security directly (by increasing food availability and accessibility) and indirectly (as a driver of economic development). In order to enable sustainable expansion of aquaculture, we need to understand aquaculture’s contribution to global greenhouse gas (GHG) emissions and how it can be mitigated. This study quantifies the global GHG emissions from aquaculture (excluding the farming of aquatic plants), with a focus on using modern, commercial feed formulations for the main species groups and geographic regions. Here we show that global aquaculture accounted for approximately 0.49% of anthropogenic GHG emissions in 2017, which is similar in magnitude to the emissions from sheep production. The modest emissions reflect the low emissions intensity of aquaculture, compared to terrestrial livestock (in particular cattle, sheep and goats), which is due largely to the absence of enteric CH_4_ in aquaculture, combined with the high fertility and low feed conversion ratios of finfish and shellfish.

## Introduction

Global aquaculture makes an important contribution to food security directly (by increasing food availability and accessibility) and indirectly (as a driver of economic development). Importantly, fish are rich in protein and contain essential micronutrients which cannot easily be substituted by other food commodities^1^.

**Figure 1:**
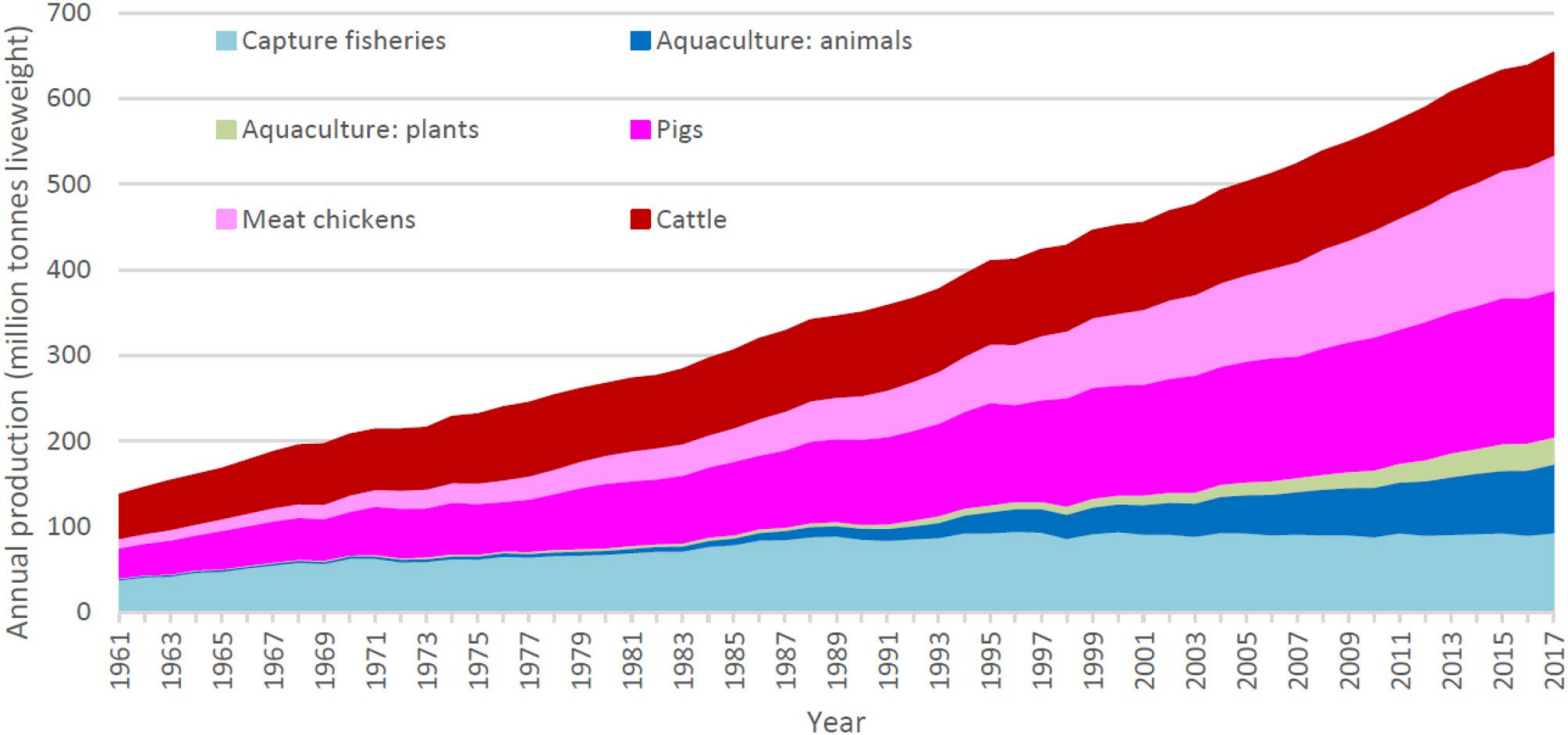
World production of capture fisheries, aquaculture and pig, chicken and cattle meat from 1961 to 2017. Data sources^14,31^.

Animal aquaculture production has expanded since the 1980s (Fig. 1) and it has been argued that the capacities for further expansion of marine aquaculture are theoretically huge^2^. In light of this, FAO^1^ concluded that as the sector further expands, intensifies and diversifies, it should recognize the relevant environmental and social concerns (e.g. competition for land and water, impacts arising from feed production, water pollution, antimicrobial resistance) and make conscious efforts to address them in a transparent manner, backed with scientific evidence.

One of the key environmental (and social) concerns is climate change, more specifically the greenhouse gas (GHG) emissions that arise along food supply chains. In order to enable sustainable expansion of aquaculture, we need to understand aquaculture’s contribution to global GHG emissions and how they can be mitigated. Here, we apply a method for quantifying the GHG emissions arising from the culture of the main aquatic animals reared for human consumption, i.e.: bivalves, shrimps/prawns and finfish (catfish, cyprinids, Indian major carps, salmonids and tilapias). The method quantifies the main GHG emissions arising “cradle to farm-gate”, from the following activities: the production of feed raw materials; processing and transport of feed materials; production of compound feed in feed mills and transport to the fish farm; rearing of fish in water. We quantify the total GHG emissions from global aquaculture and compare these emissions with other livestock sectors. We also calculate the emissions intensity (i.e. the kg of GHG emissions per unit of edible output) of aquaculture and explain the factors that influence it. Importantly, we have used recent commercial feed formulations for the main species groups and geographic regions, thereby providing a more up to date and detailed analysis than is generally provided in academic literature.

## Results

### Total emissions from global aquaculture

We calculated the GHG emissions for the year 2017 for the nine major aquaculture culture groups (which accounted for 93% of global aquaculture production, Table 1). The total GHG emissions for this 93% were 245 MtCO_2_e (Table 2). Assuming that the remaining 7% of production has the same emissions intensity (EI), the total emissions in 2017 for all shellfish and finfish aquaculture would be 263 MtCO2e. UNEP^3^ estimated total anthropogenic emissions to be 53.5GtCO2eq/year in 2017, so the culture of aquatic animals represented approximately 0.49% of total anthropogenic emissions (i.e. 263Mt/53.5Gt).

**Table 1:** Production of different culture groups by region, 2017. Source^14^.

	Bivalves	Catfish	Cyprinids	Freshwater fish, general	Indian major carps	Marine fish, general	Salmonids	Shrimps and prawns	Tilapias	Total
**Production (thousand tonnes of liveweight)**										
East Asia	14,567	4,011	21,428	3,743	0	3,937	0	6,172	3,774	57,633
South Asia	0	1,066	1,199	946	4,362	0	0	829	0	8,401
Sub-Saharan Africa	0	241	29	59	0	0	0	0	248	577
West Asia & Northern Africa	0	0	216	0	0	508	123	0	989	1836
Central & South America	396	0	0	229	0	0	939	792	509	2,864
Oceania	117	0	0	0	0	19	68	0	0	204
Eastern Europe	0	0	111	0	0	0	23	0	0	134
Western Europe	622	0	0	0	0	0	1,803	0	0	2,425
North America	212	150	0	0	0	0	185	65	0	612
Russian Federation	0	0	119	0	0	0	51	0	0	170
WORLD	15,914	5,468	23,102	4,977	4,362	4,465	3,191	7,857	5,520	74,855

**Table 2:** GHG emissions by culture group and region, 2017, calculated in this study.

	Bivalves	Catfish	Cyprinids	Freshwater fish, general	Indian major carps	Marine fish, general	Salmonids	Shrimps and prawns	Tilapias	Total
**GHG emissions (thousand tonnes of CO**_**2**_**e)**										
East Asia	16,775	13,018	70,264	13,468	0	20,695	0	43,782	15,319	193,319
South Asia	0	2,788	3,763	3,144	12,743	0	0	5,270	0	27,708
Sub-Saharan Africa	0	530	74	160	0	0	0	0	812	1,576
West Asia & Northern Africa	0	0	592	0	0	2,203	263	0	3,288	6,346
Central and South America	389	0	0	522	0	0	4,215	2,418	1,017	8,561
Oceania	126	0	0	0	0	215	133	0	0	474
Eastern Europe	0	0	176	0	0	0	49	0	0	225
Western Europe	639	0	0	0	0	0	4,902	0	0	5,542
North America	228	356	0	0	0	0	420	295	0	1,299
Russian Federation	0	0	189	0	0	0	119	0	0	307
WORLD	18,157	16,692	75,057	17,294	12,743	23,112	10,102	51,764	20,436	245,357

The geographical pattern of emissions closely mirrors production, i.e. most of the emissions arise in the regions with the greatest production: East Asia and South Asia. Emissions also correlate closely with production for most species-groups, e.g. cyprinids account for 31% of emissions and 31% of production. However, there are exceptions to this: shrimp account for 21% of emissions but only 10% of production, while bivalves produce 7% of emissions but represent 21% of production.

**Figure 2:**
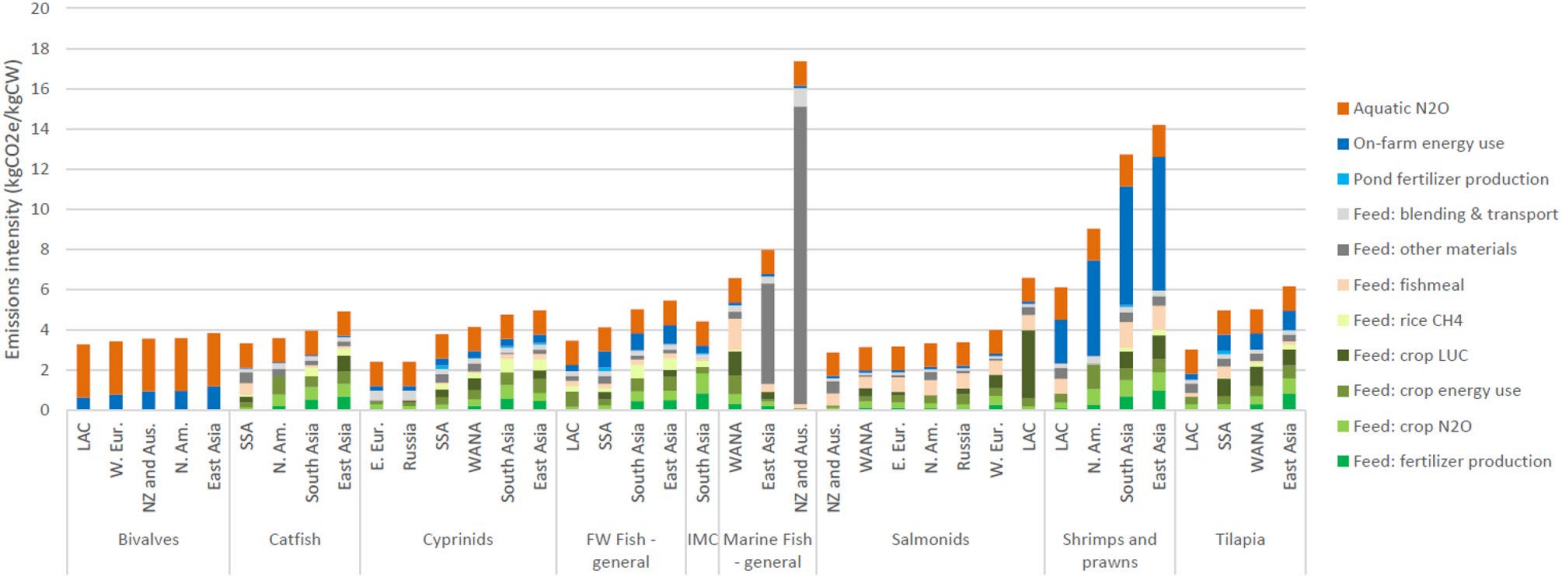
Emissions intensity of the main aquaculture groups, 2017. *Source* calculated in this study. *IMC* Indian Major Carps, *E. Eur*, Eastern Europe, *LAC* Latin America and the Caribbean, *N. Am.* North America, *NZ and Aus.* New Zealand and Australia, *SSA* Sub-Saharan Africa, *W. Eur.* Western Europe, *WANA* West Asia and North Africa.

Production of crop feed materials (the green segments of Fig. 2) accounted for 39% of total aquaculture emissions. When the emissions arising from fishmeal production, feed blending and transport are added, feed production accounts for 57% of emissions. The bulk of the non-feed emissions arise from the nitrification and denitrification of nitrogenous compounds in the aquatic system (“aquatic N_2_O”) and energy use on the fish farm (primarily for pumping water, lighting and powering vehicles).

**Figure 3:**
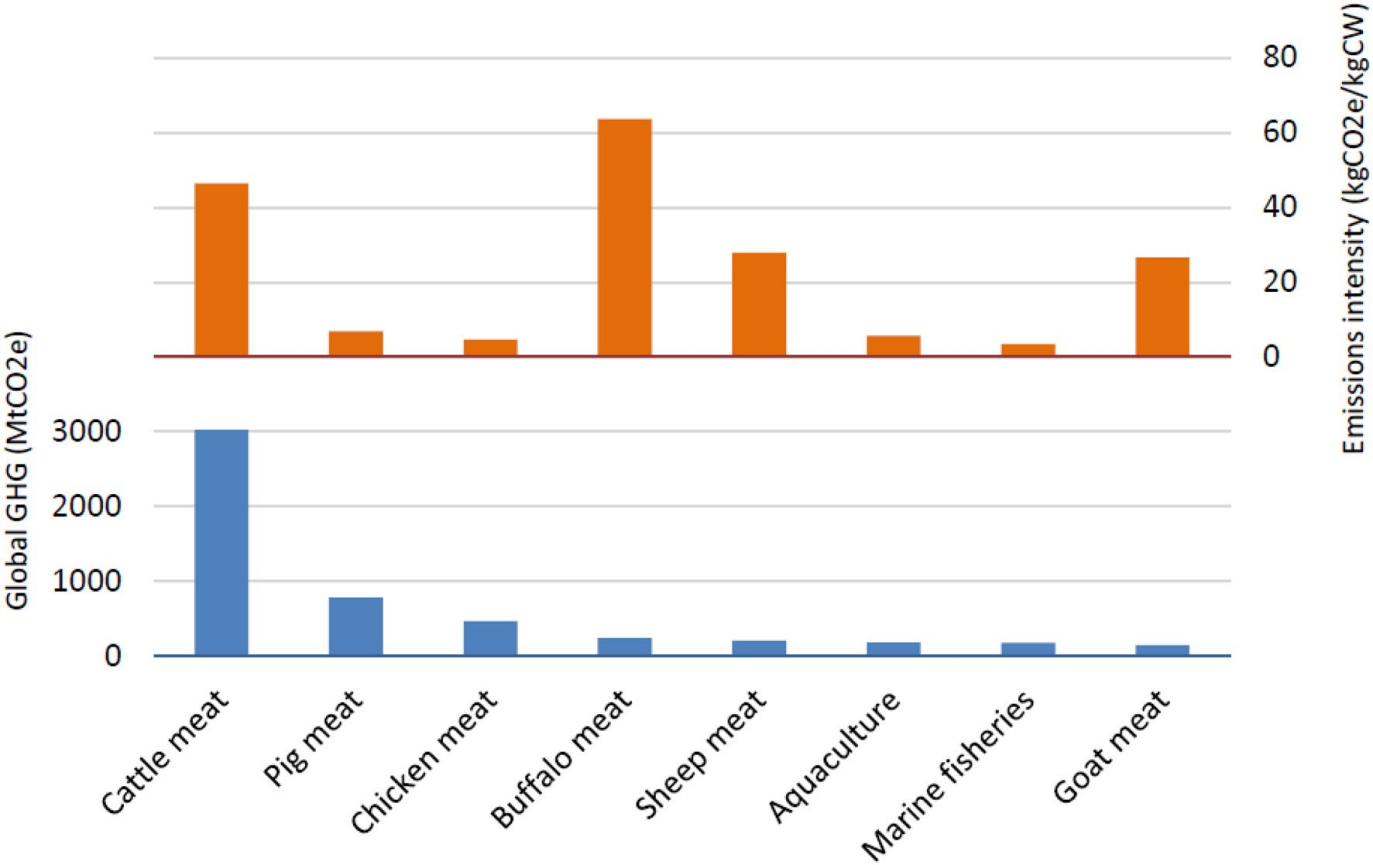
Total global emissions and emissions intensity of aquaculture (2010), terrestrial meat (2010) and marine fisheries (2011). Sources: Aquaculture—calculated in this study. Marine fisheries^5^. Cattle, pig, chicken, buffalo, sheep and goat meat^4^.

In order to compare aquaculture emissions with those arising from meat production, the aquaculture emissions for 2010 were calculated and compared with the emissions calculated for livestock by FAO using GLEAM^4^. The FAO data^4^ were used for comparison as they have the same scope and methods as the method used in this paper. 2010 was chosen as it is the most recent year for which FAO have reported global livestock results. The results of the comparison are presented in Fig. 3. This figure also includes the global emissions for capture fisheries^5^ though these are for 2011 rather than 2010.

The global emissions from aquaculture are lower than livestock because (a) there is a greater amount of livestock production (in 2010 fish and shellfish accounted for 6% of global protein intake, compared to 18% of protein from meat^6^) and (b) overall livestock has a higher emissions intensity than aquaculture.

### Emissions intensity of aquaculture

The regional average EI of each species-group is shown in Fig. 2. For most of the finfish, the EI lies between 4 and 6 kgCO_2_e/kg CW (carcass weight, i.e. per kg of edible flesh) at the farm gate. The exception is the category “marine fishes, general”, which has a significantly higher EI, due to the assumption that the ration in East Asia (and New Zealand and Australia) is 100% low value fish/trash fish (which has a higher EI than most crop feed materials) and the higher feed conversion ratio (FCR, i.e. the kg of feed input per unit of liveweight gain) of this species-group. Shrimps and prawns have high EI, due to the greater amounts of energy used in these systems (primarily for water aeration and pumping). In contrast, bivalves have the lowest EI as they have no feed emissions, relying on natural food from their environment. Within the finfish, there are some differences in the sources of GHG emissions. Species predominantly reared in Asia (i.e. Indian major carps, freshwater catfishes and cyprinids) have higher rice methane emissions, while the carnivorous salmonids have more emissions associated with fishmeal and higher crop land use change (LUC) emissions (arising from soybean production), reflecting their higher protein rations.

Comparing global averages, aquaculture has a much lower EI than ruminant meat and is similar to the main monogastric commodities (pig meat and broiler meat) (Fig. 3). It should be noted that there can be significant variation in the EI of commodities, depending on factors such as genetics, feeding and farm management (for a discussion of the factors influencing the EI of ruminants and monogastrics, see^7,8^). Fish (both finfish and shellfish) have lower EI than ruminants for three main reasons: they do not produce CH_4_ via enteric fermentation, they have much higher fertility (so the “breeding overhead” is therefore much lower) and they have lower feed conversion ratios (which are a key determinant of fish EI, given the predominance of feed related emissions). Fish generally have lower FCRs than terrestrial mammals, due to the latter’s higher maintenance and respiratory costs^9^. Being buoyant and streamlined, fish require less energy for locomotion, they are cold-blooded, and they excrete ammonia directly.

## Discussion

### Limitations of the analysis

The emissions are calculated for aquaculture of aquatic animals only, and therefore do not include the emissions arising from the production of aquatic plants, which constitute a significant proportion of global aquaculture production.

The importance of feed is clear in Fig. 2 for all fed species. However, feed composition is constantly changing as nutritional knowledge and its application develop in response to commercial demand. This study was based on regional assumptions of feed formulations and raw material origins for the main species in the key regions. Data for this was obtained from a variety of sources (see “Methods”) and updated in light of discussions with feed companies. Improved knowledge of feed formulation and raw material sourcing, combined with the overall feed efficiencies of conversion to edible seafood will help provide a more accurate picture of the overall emissions. Ultimately this would have to be done with primary data from feed companies and farmers on a case by case level.

The analyses do not include losses and emissions occurring post-farm. Depending on the specifics of the post-farm supply chain (e.g. mode of transport, distance transported, mode of processing, storage conditions), significant emissions can arise from energy use in transportation or from refrigerant leakage in cold chains^10^. However, it should be noted that all GHG emissions are attributed to the aquaculture in this study, whereas, in practice, aquaculture produces processing by-products (such as trimmings) that are often used in other sectors and the associated emissions should be allocated to these sectors.

The estimates of aquatic N_2_O should be treated with caution, as the rate at which N is converted to N_2_O in aquatic systems can vary greatly, depending on the environmental conditions. It has been noted^11^ that nitrification and denitrification processes are influenced by many parameters (e.g. dissolved oxygen concentration, pH, temperature).

Finally, this study relies on data currently available in the literature. While the best available data has been used, we recommend that true empirical studies, involving primary data gathering on key parameters, should be undertaken to validate the results.

### Reducing emissions from aquaculture

It has been argued^12^ that because the aquaculture sector is relatively young compared with terrestrial livestock sectors, it offers great scope for technical innovation to further increase resource efficiency. They go on to identify four broad technological approaches to reducing the environmental impact of aquaculture: (1) breeding and genetics, (2) disease control, (3) nutrition and feeding, and (4) low-impact production systems. Within each of these approaches are many individual measures that could be used to reduce (or mitigate) GHG emissions.

There are many ways of reducing emissions from crop production^13^ that could be employed to reduce aquaculture feed emissions. Other measures to reduce feed emissions target the efficiency of feeding. Aquaculture nutrition is arguably more complicated than terrestrial livestock production, in the sense that it has many more species being farmed. Each species in theory has different nutritional requirements, although the information to provide this accurately is often lacking. This drives relatively poor use of nutrients as the focus is instead on providing certain raw materials that mimic what is consumed in the wild—for example feeding high inclusions of fishmeal to some carnivorous species, in particular marine fishes. The opportunity to optimise nutrition is probably greater in aquaculture than in terrestrial species, since much greater research effort has been focussed on terrestrial species to date.

Some mitigation measures may be quite expensive while others are relatively cheap or may even reduce costs. In order to achieve the twin goals of reducing emissions, while increasing the supply of affordable protein, we need to analyse the effects that introducing measures may have on farm profits and emissions. Cost-effectiveness analysis (CEA) can help us to understand these effects.

## Conclusions

Aquaculture is a biologically efficient way of producing animal protein compared to terrestrial livestock (particularly ruminants) due largely to the high fertility and low feed conversion ratios of fish. The biological efficiency is reflected in the relatively low prices and emissions intensities of many aquaculture commodities. However, the moderate GHG emissions from aquaculture should not be grounds for complacency. Aquaculture production is increasing rapidly, and emissions arising post-farm, which are not included in this study, could increase the emissions intensity of some supply chains significantly. Furthermore, aquaculture can have important non-GHG impacts on, for example, water quality and marine biodiversity. It is therefore important to continue to improve the efficiency of global aquaculture to offset increases in production so that it can continue to make an important contribution to food security. Fortunately, the relatively immature nature of the sector (compared to agriculture) means that there is great scope to improve resource efficiency through technical innovation, often in ways that reduce emissions while improving profitability. CEA can be used to help identify the most cost-effective efficiency improvements, thereby supporting the sustainable development of aquaculture.

## Methods

### Scope

The system boundary is “cradle to farm-gate”. It is recognised that significant emissions (and losses of product) can occur post-farm during transport, processing and distribution.

Global aquaculture is a complex sector consisting of many different species reared in a variety of systems and environments. In order to manage this complexity, the analysis focuses on the main cultured aquatic animal species-groups (aquatic plants are excluded), i.e.: bivalves, catfish, cyprinids, freshwater fish (general), Indian major carps, marine fish (general), salmonids, shrimps and prawns and tilapias. The main species-groups were identified by extracting production data from FAO^14^, listing the species-groups within each geographical region (according to FAO definitions) in order of production amount, then selecting the groups until they accounted for > 90% of the production within the region (> 85% in Eastern Europe). This approach captured an estimated 93% of global production. The categories of GHG included in the analysis are summarised in Table 3.

**Table 3:** Summary of the GHG categories included in the calculations.

Name	Description
Feed: fertilizer production	Emissions arising from the production of synthetic fertilizers applied to crops
Feed: crop N_2_O	Direct and indirect nitrous oxide from the application of N (synthetic and organic) to crops and crop residue management
Feed: crop energy use	CO_2_ from energy use in field operations, feed transport and processing
Feed: crop LUC	CO_2_ from land use change arising from soybean cultivation
Feed: rice CH_4_	Methane arising from flooded rice cultivation
Feed: fishmeal	CO_2_ from energy use in the production of fishmeal
Feed: other materials	Emissions from the production of a small number of "other" feeds (including animal by-products, lime and synthetic amino acids)
Feed: blending and transport	CO_2_ from energy use in the production and distribution of compound feed
Pond fertilizer production	Emissions arising from the production of synthetic fertilizers applied to increase aquatic primary productivity
On-farm energy use	Emissions arising from the use of electricity and fuels on fish farm
Aquatic N_2_O	N_2_O from the microbial transformation of nitrogenous materials (fertilizers, excreted N and uneaten feed) in the fish farm water body

### Carbon sequestration in pond sediments

Carbon sequestration in pond sediments is not included in this study. While it has been suggested^15,16^ that ponds could act as net carbon sinks, questions have been raised about the certainty of the abatement rate and permanence of the sequestration^17^. There are also concerns about impacts on water quality and fish health arising from the nutrient inputs associated with carbon sequestration^18^.

### Calculation method

**Figure 4:**
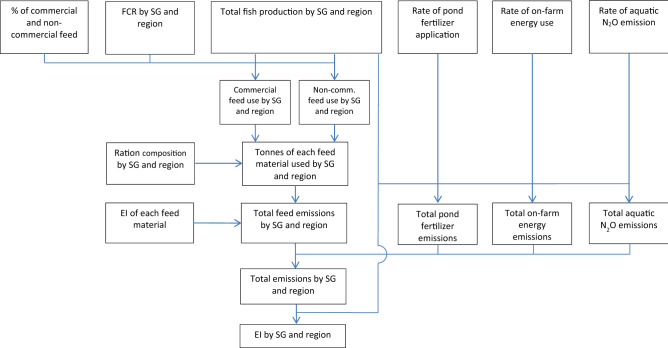
Schematic diagram of the method used to quantify the total emissions and emissions intensity. *SG* species-group; *FCR* feed conversion ratio; *EI* emissions intensity.

The method is summarised in Fig. 4 and further details are provided below.

### Emission factors for feed raw materials

The emission factors (EFs) for crop feed materials were based on the values derived using GLEAM^4^. Regional average values were used for each feed, meaning that the EFs at least partially capture variation in crop production efficiency between regions. EFs for additional feeds (e.g. fishmeal, poultry meal, feather meal, meat & bone meal, blood meal, groundnut meal) were derived from Feedprint^19^ and EFs for fish oil from^20^. Non-commercial feed materials were assumed to be produced locally, and have different emission profiles to their commercial equivalents (e.g. no emissions from transport).

### Emission factors for fertilizers

EFs for fertilizers such as urea and potash were derived from^21^, which provides EFs for each fertilizer for five geographic regions: western Europe; Russian Federation and central Europe; North America; China and India; and rest of the world.

### Feed conversion ratios and ration composition

A distinction was made between two types of aquafeed as follows: (a) commercial aquafeed, which are compound feeds purchased from specialised feed manufacturers and/or feed wholesalers/retailers. The feed is comprised of materials sourced nationally and internationally, which are formulated and blended into high quality compounded pellet feeds and (b) farm-made/semi-commercial aquafeeds (which often include mashes or wet pellets) made on the farm or produced by small-scale feed manufacturers from locally sourced feed materials. The proportions of production reared on commercial and non-commercial rations were estimated based on^22^.

Changes in commercial conditions make it difficult to keep up to date through academic papers, as the feed compositions are improved/changed frequently and farming conditions fluctuate with improvements and emerging disease challenges. To account for this, feed composition (protein and energy), raw material rations and economic feed conversion ratios (eFCRs, which take into account average mortalities) were derived from a range of sources including: AFFRIS^23^, FAO publications^24–26^, journal articles (e.g.^22,27^), grey literature (e.g.^28^) and expert opinion to reflect the most recent updates. Feed conversion ratios used and the ration assumptions for the main culture group x location combinations are given in the Supplementary Information.

### Total production by species-group and region

Production data for 2017 was extracted from the FAO database FishStatJ^14^.

### On-farm energy use

Energy is used on fish farms for a variety of purposes, primarily for pumping water, lighting and powering vehicles. The average amount of energy required to produce one tonne of live weight of fish and shellfish, and the proportions of electricity, diesel and petrol used, were calculated based on values presented in the literature (see Supplementary Information). The rates of electricity, diesel and petrol used per tonne of live weight (LW) were then multiplied by emission factors to determine the emission intensity (see Supplementary Information). Global EFs were used for petrol and diesel, and regional EFs were used for grid electricity ^29^.

### Aquatic N_2_O emissions

According to^11^ N_2_O emissions from the water body on the fish farm arise “from the microbial nitrification and denitrification, the same as in terrestrial or other aquatic ecosystems”. However, quantifying the emissions from the pond surface to the air is challenging, because they depend on the pH and dissolved oxygen content of the pond, and both fluctuate greatly^30^. Despite these difficulties, pond N_2_O emissions were included in the present study, to illustrate their likely contribution to the total emissions, and to allow the comparison of the GHG associated with aquaculture products to be compared with the GHG associated with terrestrial livestock products (for which N_2_O from excreted N is routinely quantified).

The amount of N_2_O per species-group was determined by multiplying the production by the N_2_O emission factor per kg of production^11^, i.e. 1.69 gN_2_O–N per kg of production, or 0.791 kgCO_2_e/kgLW production. This equates to a conversion rate of N to N_2_O–N of 1.8%, which is higher than the 0.71% used in^17^.

## Supplementary information

Supplementary file1 (DOCX 164 kb)

## References

[1] FAO. *World Aquaculture 2015: A Brief Overview* 34 (FAO Fisheries and Aquaculture Circular No. 1140, 2017).

[2] Gentry RR (2017). Mapping the global potential for marine aquaculture. Nat. Ecol. Evol..

[3] UNEP (2018). The Emissions Gap Report 2018.

[4] FAO. *Global Livestock Environmental Assessment Model (GLEAM)* 109 (FAO, Rome, 2017) www.fao.org/gleam/en/.

[5] Parker RWR (2018). Fuel use and greenhouse gas emissions of world fisheries. Nat. Clim. Change.

[6] FAO. *FAOSTAT Food Balance Sheets*. www.fao.org/faostat/en/#data/FBS. (2017).

[7] Opio C (2013). Greenhouse Gas Emissions from Ruminant Supply Chains: A Global Life Cycle Assessment.

[8] MacLeod M (2013). Greenhouse Gas Emissions from Pig and Chicken Supply Chains: A Global Life Cycle Assessment.

[9] Gjedrem T, Robinson N, Rye M (2012). The importance of selective breeding in aquaculture to meet future demands for animal protein: a review. Aquaculture.

[10] Winther, U. *et al*. *Carbon footprint and energy use of Norwegian seafood products* 89 (SINTEF Fisheries and Aquaculture Report No. SFH80 A096068, Trondheim, 2009).

[11] Hu Z, Lee JW, Chandran K, Kim S, Khanal SK (2012). Nitrous oxide (N_2_O) emission from aquaculture: a review. Environ. Sci. Technol..

[12] Waite, R. *et al*. *Improving Productivity and Environmental Performance of Aquaculture.* Working Paper, Instalment 5 of “Creating a Sustainable Food Future 59 (World Resources Institute, Washington, DC, 2014).

[13] MacLeod, M., Eory, V., Gruere, G., & Lankoski, J. *Cost-Effectiveness of Greenhouse Gas Mitigation Measures for Agriculture: A Literature Review* 73 (OECD Food, Agriculture and Fisheries Papers, No. 89. 73 (OECD Publishing, Paris, 2015).

[14] FAO. *FishStatJ, Vers. 3.01.0* (FAO, Rome, 2019). www.fao.org/fishery/statistics/software/fishstatj/en.

[15] Verdegem MCJ, Bosma RH (2009). Water withdrawal for brackish and inland aquaculture, and options to produce more fish in ponds with present water use. Water Policy.

[16] Boyd CE, Wood CW, Chaney PL, Queiroz JF (2010). Role of aquaculture pond sediments in sequestration of annual global carbon emissions. Environ. Pollut..

[17] Henriksson, P. J. G. et al. Final LCA Case Study Report—Results of LCA Studies of Asian Aquaculture Systems for Tilapia, Catfish, Shrimp, and Freshwater Prawn. SEAT Deliverable Ref: D 3.5 (2014).

[18] MacLeod, M., Hasan, M. R., Robb, D. H. F., & Mamun-Ur-Rashid, M. *Quantifying and Mitigating Greenhouse Gas Emissions from Global Aquaculture*. FAO Fisheries and Aquaculture Technical Paper No. 626. (FAO, Rome, 2019).

[19] Feedprint. *Feedprint Carbon Footprint of Animal Nutrition*. https://webapplicaties.wur.nl/software/feedprint/Wageningen (2017).

[20] Pelletier N, Tyedmers P (2010). A life cycle assessment of frozen Indonesian tilapia fillets from lake and pond-based production systems. J. Ind. Ecol..

[21] Kool A, Marinussen M, Blonk H (2012). LCI Data for the Calculation Tool Feedprint for Greenhouse Gas Emissions of Feed Production and Utilization: GHG Emissions of N, P, and K Fertilizer Production.

[22] Tacon AJG, Metian M (2015). Feed matters: satisfying the feed demand of aquaculture. Rev. Fish. Sci. Aquac..

[23] AFFRIS. *Aquaculture Feed and Fertilizer Resources Information System* (FAO, Rome) www.fao.org/fishery/affris/en/ (2017).

[24] Tacon, A. G. J., Metian, M., & Hasan, M. R. 2009. *Feed Ingredients and Fertilizers for Farmed Aquatic Animals: Sources and Composition.* FAO Fisheries and Aquaculture Technical Paper. No. 540, 209 (FAO, Rome, 2009). www.fao.org/docrep/012/i1142e/i1142e.pdf.

[25] Hasan, M. R., & Soto, S. *Improving Feed Conversion Ratio and Its Impact on Reducing Greenhouse Gas Emissions in Aquaculture*. FAO Non-Serial Publication 33 (FAO, Rome, 2017). https://www.fao.org/3/a-i7688e.pdf).

[26] Robb, D. H. F, MacLeod, M., Hasan, M. R. & Soto, D. *Greenhouse gas emissions from aquaculture: a life cycle assessment of three Asian systems*. FAO Fisheries and Aquaculture Technical Paper No. 609 117 (FAO, Rome, 2017).

[27] Tacon AGJ, Metian M (2008). Global overview on the use of fishmeal and fish oil in industrially compounded aquafeeds: trends and future prospects. Aquaculture.

[28] White, A. *A Comprehensive Analysis of Efficiency in the Tasmanian Salmon industry* 301. PhD thesis, Bond University, Australia (2013).

[29] BEIS (Department for Business Energy & Industrial Strategy). *Government GHG conversion factors for company reporting: Methodology paper for emission factors* 112 (UK Department of Business, Energy & Industrial Strategy, London). https://assets.publishing.service.gov.uk/government/uploads/system/uploads/attachment_data/file/553488/2016_methodology_paper_Final_V01-00.pdf) (2016).

[30] Bosma R, Thi Anh P, Potting J (2011). Life cycle assessment of intensive striped catfish farming in the Mekong Delta for screening hotspots as input to environmental policy and research agenda. Int. J. Life Cycle Assess..

[31] FAO. *FAOSTAT Production Data* www.fao.org/faostat/en/#data. (2020).

