# Supplementary material for: Quantifying greenhouse gas emissions from global aquaculture
Source: Sci Rep. 2020 Jul 15;10:11679. doi: 10.1038/s41598-020-68231-8 (PMC7363927; doi:10.1038/s41598-020-68231-8)
Supplement: Supplementary file 1 — Supplementary file1 (DOCX 164 kb) [file 41598_2020_68231_MOESM1_ESM.docx]

**Supplementary Information:** Quantifying greenhouse gas emissions from global aquaculture

Michael J. MacLeod^a*^, Mohammad R. Hasan^b^, David H.F. Robb^c^ and Mohammad Mamun-Ur-Rashid^d^

1. Rural Economy, Environment and Society Group, SRUC, Edinburgh, United Kingdom
2. Aquaculture Branch, FAO Fisheries and Aquaculture Department, Rome, Italy
3. Cargill Animal Nutrition and Health, aquaculture business, Surrey, United Kingdom
4. WorldFish, Dhaka, Bangladesh

*Corresponding author. michael.macleod@sruc.ac.uk

*System boundary*

The system boundary of the analysis is shown in Figure A. It was defined based on a review of previous studies, which indicated that the emissions intensity (EI) was likely to be primarily a function of processes occurring during the following stages:

• Production of feed raw materials;

• Processing and transport of feed materials;

• Production of compound feed in feed mills and transport to the fish farm;

• Rearing of fish in water.

*Figure A. Inputs to the aquaculture chains that may impact GHG emissions [24]. The system boundary of the study is indicated by the dashed red line*


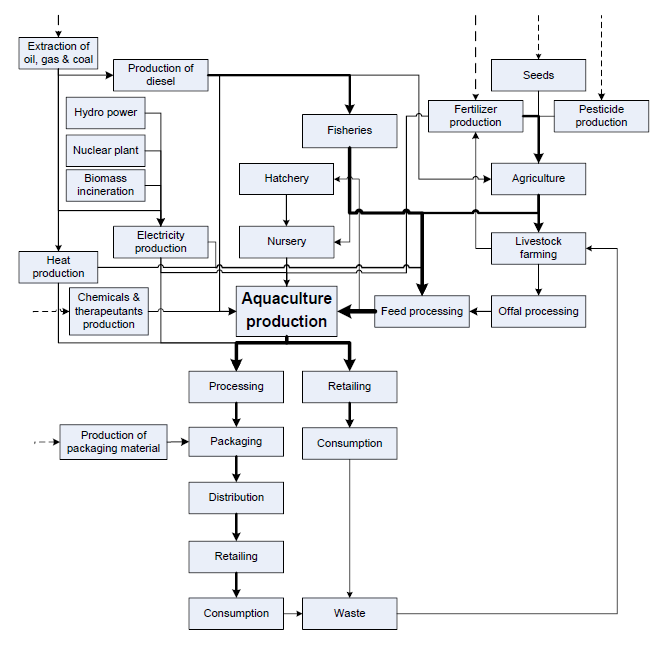


*GHG categories*

The major GHGs associated with aquaculture production are:

- **N_2_O** (nitrous oxide) arising from the microbial transformation of N (nitrogen) (mainly from applied fertilizers) in soils during the cultivation of feed crops. Significant amounts of N_2_O may also be emitted from ponds as a result of the microbial transformation of nitrogenous compounds in ponds (e.g. synthetic fertilizers, manures, composts, uneaten feed and excreted N), although the magnitudes of these emissions are less readily quantified.
- **CO_2_** (carbon dioxide) arising from *pre-farm* energy use (primarily associated with feed and fertilizer production), *on-farm* energy use (e.g. pumping of water, use of electricity, other fuel consumption) and during *post-farm* distribution and processing. CO_2_ emissions also arise from changes in above and below ground carbon stocks induced by land use and land use change (LUC) (primarily driven by increased demand for feed crops, which can lead to the conversion of forest and grassland to arable land).
- **CH_4_** (methane) arising mainly from the anaerobic decomposition of organic matter during flooded rice cultivation. May also arise during fish farm waste management.
- **F-gases** (fluorinated gases) - small amounts of these potent greenhouse gases are leaked from cooling systems on-farm and post-farm.

GHG sources falling within the cradle to farm-gate system boundary, but not included in the analysis, are summarised in Table A.

*Table A. GHG sources falling within the cradle to farm-gate system boundary, but not included in the analysis*

| **Process** | **Gas** | **Comment** |
| --- | --- | --- |
| Energy in the manufacture of on-farm buildings and equipment (including packaging) | CO_2_ | Difficult to quantify, unlikely to be a major source of emissions |
| Production of cleaning agents, antibiotics and pharmaceuticals | CO_2_ | Unlikely to be a major source of emissions |
| Anaerobic decomposition of organic matter in ponds | CH_4_ | Difficult to quantify, unlikely to be a major source of emissions |
| N_2_O from the animal | N_2_O | Possibly significant for invertebrates, but difficult to quantify |
| LUC arising from pond construction | CO_2_ | Difficult to quantify, unlikely to be a major source of emissions |
| Pond cleaning maintenance | CO_2_ | Difficult to quantify, unlikely to be a major source of emissions |
| CO_2_ sequestered in carbonates | CO_2_ | Possibly significant for invertebrates? |
| CO_2_ sequestered in pond sediments | CO_2_ | Difficult to quantify, potentially significant |
| Leakage of coolants | F-gases | Difficult to quantify, potentially significant (particularly post-farm) |

*Feed assumptions*

Feed conversion ratios used and their sources are given in Table B, and the ration assumptions for the main culture group x location combinations are given in Table C.

*Table B. Feed conversion ratios used in the model as representative of the species-group in each region, for systems using commercial feed. FCR is calculated from the kg of feed (as fed) that is used to produce 1 kg of live fish; (-) indicates species-group x location combination not included in the study. [a] indicates a value based on personal observation.*

| **Region** | East and Southeast Asia | | South Asia | | Sub-Saharan Africa | | West Asia & North Africa | | Latin America and Caribbean | | New Zealand and Australia | | Eastern Europe | | Western Europe | | North America | | Russian Federation | |
| --- | --- | --- | --- | --- | --- | --- | --- | --- | --- | --- | --- | --- | --- | --- | --- | --- | --- | --- | --- | --- |
| **Species-group** | FCR | *Sources* | FCR | *Sources* | FCR | *Sources* | FCR | *Sources* | FCR | *Sources* | FCR | *Sources* | FCR | *Sources* | FCR | *Sources* | FCR | *Sources* | FCR | *Sources* |
| Catfish (freshwater) | 1.69 | [8] | 1.69 | [8] | 1.20 | [4,13] | - |  | - |  | - |  | - |  | - |  | 2.50 | [18] | - |  |
| Cyprinids | 1.70 | [4, 9, 10, 11, 12] | 1.80 | [4, 8, 9,10, 11, 12] | 1.80 | [4] | 1.70 | [10,11, 12] | - |  | - |  | 1.70 | [a] | - |  | - |  | 1.80 | [4,10, 11,12] |
| Freshwater fish, general | 1.80 | [8] | 1.80 | [8] | 1.80 | [4] | - |  | 1.80 | [4] | - |  | - |  | - |  | - |  | - |  |
| Indian major carps | - |  | 1.80 | [8] | - |  | - |  | - |  | - |  | - |  | - |  | - |  | - |  |
| Marine fish, general | 1.70 | [a] | - |  | - |  | 2.75 | [4] | - |  | 1.52 | [3] | - |  | 2.06 | [14,15, 16,17] | - |  | - |  |
| Salmonids | - |  | - |  | - |  | 0.92 | [4,5] | 1.30 | [4,6] | 1.41 | [1,2,3] | 1.20 | [a] | 1.13 | [6,7] | 1.30 | [6] | 1.25 | [a] |
| Shrimps and prawns | 1.91 | [a] | 1.83 | [a] | - |  | - |  | 1.50 | [a] | - |  | - |  | - |  | 2.48 | [a] | - |  |
| Tilapias | 1.70 | [8] | 1.59 | [8] | 1.70 | [4] | 1.70 | [4] | 1.70 | [4] | - |  | - |  | - |  | - |  | - |  |

*Table C. Rations for the main culture group x location combinations (accounting for 90% of total feed input into global aquaculture). Abbreviations: C – commercial feed ration; NC – non-commercial feed ration; LAC – Latin America and the Caribbean; WANA – West Asia and North Africa; DDGS – dried distillers’ grains with solubles.*

|  | | | *Non-local crop feed materials* | | | | | | | | | *Local crop feed materials* | | | | | | | | | | | | *Other feed materials* | | | | | | |
| --- | --- | --- | --- | --- | --- | --- | --- | --- | --- | --- | --- | --- | --- | --- | --- | --- | --- | --- | --- | --- | --- | --- | --- | --- | --- | --- | --- | --- | --- | --- |
| *Culture group* | *Region* | *Feed type* | Soybean meal | Soybean oil | Oilseed meal | Wheat | Wheat flour | Wheat by-products | Maize | Maize gluten meal | Rice by-products | Soybean, whole | Soybean meal | Oilseed meal | Oilseed oil | Wheat flour | Wheat by-products | Wheat | Maize | Maize gluten meal | Maize DDGS | Rice by-products | Cassava | Groundnut meal | Fishmeal - reduction | Fish oil - reduction | Poultry meal | Meat and bone meal | Vitamin and mineral premix | Low-value fish |
| Catfish | East Asia | C | 25% | 0% | 12% | 7% | 0% | 6% | 0% | 0% | 0% | 0% | 0% | 0% | 0% | 0% | 0% | 0% | 0% | 0% | 0% | 21% | 18% | 0% | 1% | 0% | 0% | 10% | 0% | 0% |
|  | East Asia | NC | 0% | 0% | 0% | 0% | 0% | 0% | 0% | 0% | 0% | 0% | 0% | 8% | 0% | 2% | 2% | 0% | 1% | 0% | 0% | 74% | 0% | 3% | 7% | 0% | 0% | 3% | 0% | 0% |
|  | South Asia | C | 0% | 0% | 0% | 0% | 0% | 0% | 0% | 0% | 0% | 0% | 25% | 12% | 0% | 0% | 13% | 0% | 0% | 0% | 0% | 21% | 18% | 0% | 1% | 0% | 0% | 10% | 0% | 0% |
| Cyprinids | East Asia | C | 20% | 0% | 10% | 0% | 0% | 0% | 0% | 0% | 30% | 0% | 0% | 0% | 0% | 0% | 0% | 0% | 0% | 0% | 0% | 0% | 25% | 0% | 5% | 0% | 0% | 10% | 0% | 0% |
|  | East Asia | NC | 0% | 0% | 0% | 0% | 0% | 0% | 0% | 0% | 0% | 0% | 0% | 8% | 0% | 2% | 2% | 0% | 1% | 0% | 0% | 74% | 0% | 3% | 7% | 0% | 0% | 3% | 0% | 0% |
|  | South Asia | NC | 0% | 0% | 0% | 0% | 0% | 0% | 0% | 0% | 0% | 0% | 0% | 8% | 0% | 2% | 2% | 0% | 1% | 0% | 0% | 74% | 0% | 3% | 7% | 0% | 0% | 3% | 0% | 0% |
| Freshwater fish, general | East Asia | C | 23% | 0% | 21% | 4% | 10% | 0% | 4% | 0% | 0% | 0% | 0% | 0% | 0% | 0% | 0% | 0% | 0% | 0% | 0% | 23% | 0% | 0% | 5% | 0% | 4% | 2% | 4% | 0% |
|  | East Asia | NC | 0% | 0% | 0% | 0% | 0% | 0% | 0% | 0% | 0% | 0% | 0% | 8% | 0% | 2% | 2% | 0% | 1% | 0% | 0% | 74% | 0% | 3% | 7% | 0% | 0% | 3% | 0% | 0% |
|  | South Asia | NC | 0% | 0% | 0% | 0% | 0% | 0% | 0% | 0% | 0% | 0% | 0% | 8% | 0% | 2% | 2% | 0% | 1% | 0% | 0% | 74% | 0% | 3% | 7% | 0% | 0% | 3% | 0% | 0% |
| Indian major carps | South Asia | C | 0% | 0% | 0% | 0% | 0% | 0% | 0% | 0% | 0% | 29% | 0% | 15% | 0% | 0% | 0% | 0% | 15% | 1% | 2% | 34% | 0% | 1% | 0% | 2% | 0% | 1% | 0% | 0% |
| Marine fish, general | East Asia | C | 30% | 0% | 0% | 16% | 0% | 0% | 0% | 0% | 0% | 0% | 0% | 0% | 0% | 0% | 0% | 0% | 0% | 0% | 0% | 0% | 0% | 0% | 32% | 8% | 0% | 10% | 4% | 0% |
|  | East Asia | NC | 0% | 0% | 0% | 0% | 0% | 0% | 0% | 0% | 0% | 0% | 0% | 0% | 0% | 0% | 0% | 0% | 0% | 0% | 0% | 0% | 0% | 0% | 0% | 0% | 0% | 0% | 0% | 100% |
| Salmonids | LAC | C | 21% | 15% | 0% | 13% | 0% | 1% | 0% | 4% | 0% | 0% | 0% | 0% | 0% | 0% | 0% | 0% | 0% | 0% | 0% | 0% | 0% | 0% | 18% | 10% | 8% | 6% | 5% | 0% |
|  | W. Europe | C | 25% | 0% | 0% | 0% | 0% | 0% | 0% | 0% | 0% | 0% | 0% | 7% | 19% | 0% | 7% | 11% | 0% | 0% | 0% | 0% | 0% | 0% | 18% | 11% | 0% | 0% | 2% | 0% |
| Shrimps and prawns | East Asia | C | 28% | 0% | 0% | 28% | 0% | 3% | 0% | 0% | 0% | 0% | 0% | 0% | 0% | 0% | 0% | 0% | 0% | 0% | 0% | 2% | 0% | 0% | 32% | 2% | 0% | 0% | 5% | 0% |
|  | East Asia | NC | 0% | 0% | 0% | 0% | 0% | 0% | 0% | 0% | 0% | 0% | 0% | 8% | 0% | 2% | 2% | 0% | 1% | 0% | 0% | 74% | 0% | 3% | 7% | 0% | 0% | 3% | 0% | 0% |
|  | South Asia | C | 28% | 0% | 0% | 28% | 0% | 3% | 0% | 0% | 0% | 0% | 0% | 0% | 0% | 0% | 0% | 0% | 0% | 0% | 0% | 2% | 0% | 0% | 32% | 2% | 0% | 0% | 5% | 0% |
| Tilapias | East Asia | C | 23% | 0% | 21% | 4% | 10% | 0% | 4% | 0% | 0% | 0% | 0% | 0% | 0% | 0% | 0% | 0% | 0% | 0% | 0% | 23% | 0% | 0% | 5% | 0% | 4% | 2% | 4% | 0% |
|  | WANA | C | 35% | 0% | 3% | 0% | 0% | 0% | 0% | 0% | 0% | 0% | 0% | 3% | 0% | 0% | 0% | 0% | 25% | 0% | 0% | 22% | 0% | 0% | 4% | 0% | 0% | 5% | 4% | 0% |

*On-farm energy use*

Energy is used on fish farms for a variety of purposes, primarily for pumping water, lighting and powering vehicles. The average amount of energy required to produce one tonne of live weight of fishes and shellfishes, and the proportions of electricity, diesel and petrol used, were calculated based on values presented in the literature (Table D). The rates of electricity, diesel and petrol used per tonne of live weight (LW) were then multiplied by emission factors (Table E) to determine the emission intensity (Table F). Global EFs were used for petrol and diesel, and regional EFs were used for grid electricity (Table E).

*Table D. Average amount of on-farm energy use to produce one tonne of live fish and shellfish and the percentage contribution of each energy source to the total. Values in the parenthesis indicates the percentage total of different energy sources*

| **Species-group** | **Average amount of on-farm energy (MJ/tLW) use** | | | | |
| --- | --- | --- | --- | --- | --- |
|  | **Electricity** | **Diesel** | **Petrol** | **Total** | **Sources** |
| Bivalves | 1 067 (37.4) | 1 790 (62.7) | 0 (0.0) | 2 857 | [23, 26] |
| Catfish (freshwater) | 206 (90.0) | 23 (10.0) | 0 (0.0) | 229 | [8, 24, 25] |
| Cyprinids | 258 (32.2) | 424 (52.9) | 119 (14.9) | 801 | [8] |
| Freshwater fish, general | 2 653 (77.0) | 586 (17.0) | 207 (6.0) | 3 446 | [8, 24, 25] |
| Indian major carps | 258 (32.2) | 424 (52.9) | 119 (14.9) | 801 | [8] |
| Marine fish, general | 0 (0.0) | 551 (47.2) | 617 (52.8) | 1 168 | [19, 20] |
| Salmonids | 0 (0.0) | 551 (47.2) | 617 (52.8) | 1 168 | [19, 20] |
| Shrimps and prawns | 14 068 (75.7) | 4 511 (24.3) | 2 (0.0) | 18 581 | [21, 22, 24, 25, 27] |
| Tilapias | 2 653 (77.0) | 586 (17.0) | 207 (6.0) | 3 446 | [8, 24, 25] |

*Table E. Energy emission factors by power type and region [28]*

| **Power type** | **Region** | **Emission factors (kgCO_2_e/MJ)** |
| --- | --- | --- |
| Diesel | Global | 0.074 |
| Petrol | Global | 0.070 |
| Electricity | North America | 0.145 |
|  | Russian Federation | 0.107 |
|  | Western Europe | 0.096 |
|  | Eastern Europe | 0.109 |
|  | West Asia & northern Africa | 0.177 |
|  | East Asia | 0.213 |
|  | New Zealand and Australia | 0.138 |
|  | South Asia | 0.186 |
|  | Latin America and Caribbean | 0.055 |
|  | Sub-Saharan Africa | 0.177 |

*Table F. Emission factor for on-farm energy use (kgCO_2_e/tLW), calculated in this study.*

|  | Bivalves | Catfish | Cyprinids | Freshwater fish, general | Indian major carps | Marine fish, general | Salmonids | Shrimps and prawns | Tilapias |
| --- | --- | --- | --- | --- | --- | --- | --- | --- | --- |
| East Asia | 360 | 46 | 267 | 623 | 267 | 84 | 84 | 3331 | 623 |
| South Asia | 331 | 40 | 238 | 551 | 238 | 84 | 84 | 2948 | 551 |
| Sub-Saharan Africa | 322 | 38 | 229 | 528 | 229 | 84 | 84 | 2826 | 528 |
| West Asia & North Africa | 322 | 38 | 229 | 528 | 229 | 84 | 84 | 2826 | 528 |
| Latin America and Caribbean | 191 | 13 | 98 | 203 | 98 | 84 | 84 | 1103 | 203 |
| New Zealand and Australia | 280 | 30 | 187 | 423 | 187 | 84 | 84 | 2272 | 423 |
| Eastern Europe | 249 | 24 | 156 | 347 | 156 | 84 | 84 | 1866 | 347 |
| Western Europe | 236 | 22 | 143 | 314 | 143 | 84 | 84 | 1692 | 314 |
| North America | 288 | 32 | 195 | 443 | 195 | 84 | 84 | 2376 | 443 |
| Russian Federation | 247 | 24 | 154 | 341 | 154 | 84 | 84 | 1834 | 341 |

**References**

[1] Walker, S.P., Symonds, J.E., Bailey, J., Dodds, K.G., Hely, F., & Amer, P. Measurement and genetics of feed conversion efficiency in three fish species. *World Aquaculture 2014* Meeting Abstract. (available at https://www.was.org/meetings/ShowAbstract.aspx?Id=33217). (2014)

[2] White, A. *A comprehensive analysis of efficiency in the Tasmanian Salmon industry*. PhD thesis, Bond University, Australia. 301 pp. (2013)

[3] Skretting Australia. *Annual Sustainability Report*. (available at https://www.skretting.com/siteassets/au-temp-files/nexus-and-reports-and-brochures/2013-austsust-report_web.pdf). (2013)

[4] Tacon, A.G.J., & Metian, M. Global overview on the use of fishmeal and fish oil in industrially compounded aquafeeds: trends and future prospects. *Aquaculture*, **285**, 146–158. (2008)

[5] Pyc, A. Expert topic - trout. *International Aquafeed*, May 16, 2012. (available at https://iaffeatures.wordpress.com/2012/05/16/expert-topic-trout/) (2012)

[6] EWOS. *Annual sustainability report.* http://www.reporting.ewos.com/sustainability-reports/gri-report-2013/performance/?id=ew-i09 (2013)

[7] Marine Harvest. *Salmon industry handbook.* (available at http://marineharvest.com/about/news-and-media/news/marine-harvest-asa-osemhg-nysemhg--2015-salmon-industry-handbook/). (2015)

[8] Robb, D.H.F, MacLeod, M., Hasan, M.R. & Soto, D. *Greenhouse gas emissions from aquaculture: a life cycle assessment of three Asian systems.* FAO Fisheries and Aquaculture Technical Paper No. 609. Rome, FAO. 110 pp. (2017)

[9] FAO. *Cultured aquatic species information program. Hypophthalmichthys nobilis* (Richardson, 1845). (available at www.fao.org/fishery/culturedspecies/Hypophthalmichthys_nobilis/en). (2016)

[10] FAO. *Aquaculture feed and fertilizer resources information system. Common carp feed formulation.* (available at www.fao.org/fishery/affris/species-profiles/common-carp/feed-formulation/en/. (2016)

[11] FAO. *Cultured aquatic species information programme. Ctenopharyngodon idellus.* (available at www.fao.org/fishery/culturedspecies/Ctenopharyngodon_idellus/en). (2016)

[12] FAO. *Aquaculture feed and fertilizer resources information system. Silver carp feed formulation*. (available at www.fao.org/fishery/affris/species-profiles/silver-carp/feed-formulation/en/). (2016)

[13] FAO. *Aquaculture feed and fertilizer resources information system. North African catfish feed formulation and manufacture.* (available at www.fao.org/fishery/affris/species-profiles/north-african-catfish/feed-formulation-and-manufacture/en/. (2016)

[14] Myrseth, B. Strategy for the production and marketing of salmon and a comparison with the seabass and seabream industry. In *Performance of the sea bass and sea bream sector in the Mediterranean*. Minutes of a workshop held within Aquaculture Europe 2014. Kursaal, Spain. 24 pp. (2010)

[15] Bjorndal, T & Fernandez-Polanco, J. *Turbot aquaculture: production and markets*. http://www.aquacultuurvlaanderen.be/sites/aquacultuurvlaanderen.be/files/public/attachments/article/871/Turbot%20aquaculture%20production%20and%20market.pdf. (2014)

[16] Ottolenghi, F. Capture-based aquaculture of bluefin tuna. In A. Lovatelli & P.F. Holthus, eds. *Capture-based aquaculture. Global overview*, pp. 169–182. FAO Fisheries Technical Paper. No. 508. Rome, FAO. 298 pp (2008)

[17] Myolonas, C.C., de la Gandara, F., Corriero, A., and Rios, A.B. Atlantic bluefin tuna (Thunnus thynnus) farming and fattening in the Mediterranean Sea. *Reviews in Fisheries Science*, **18**, 3, 266 –281 (2010)

[18] Robinson, E.H. & Li, M.H. *Feed conversion ratio for pond-raised catfish. Information sheet 1364*, Mississippi Agricultural and Forestry Experiment Station, Mississippi State University, USA. 5 p. (2015)

[19] Ayer, N.W. & Tyedmers, P.H. Assessing alternative aquaculture technologies: life cycle assessment of salmonid culture systems in Canada. *Journal of Cleaner Production*, **17**, 3, 362–373. (2008)

[20] Pelletier, N., Tyedmers, P., Sonesson, U., Scholz, A., Ziegler, F., Flysjo, A., Kruse, S., Cancino, B. & Silverman, H. Not all salmon are created equal: life cycle assessment (LCA) of global salmon farming systems. *Environmental Science & Technology*, **43**, 23, 8730–8736 (2009)

[21] Sun, W. *Life cycle assessment of indoor recirculating shrimp aquaculture system.* MSc Thesis University of Michigan. 52 pp. (2009)

[22] Cao, L. *Farming shrimp for the future: A sustainability analysis of shrimp farming in China.* PhD Thesis, University of Michigan. 160 pp. (2012)

[23] Fry, J.M. *Carbon footprint of Scottish suspended mussels and intertidal oysters.* Pitlochry, Scotland: Scottish Aquaculture Research Forum (SARF). 56 pp. (2012)

[24] Henriksson, P.J.G., Zhang, W., Nahid, S.A.A., Newton, R., Phan, L.T., Dao, H.M., Zhang, Z., Jaithiang, J., Andong, R., Chaimanuskul, K., Vo, N.S., Hua, H.V., Haque, M.M., Das, R., Kruijssen, F., Satapornvanit, K., Nguyen, P.T., Liu, Q., Liu, L., Wahab, M.A., Murray, F.J., Little, D.C. & Guinée, J.B. *Final LCA case study report—results of LCA studies of Asian aquaculture systems for tilapia, catfish, shrimp, and freshwater prawn.* SEAT Deliverable Ref: D 3.5. (2014)

[25] Henriksson, P.J.G., Zhang, W., Nahid, S.A.A., Newton, R., Phan, L.T., Dao, H.M., Zhang, Z., Jaithiang, J., Andong, R., Chaimanuskul, K., Vo, N.S., Hua, H.V., Haque, M.M., Das, R., Kruijssen, F., Satapornvanit, K., Nguyen, P.T., Liu, Q., Liu, L., Wahab, M.A., Murray, F.J., Little, D.C. & Guinée, J.B. *Final LCA case study report. Primary data and literature sources adopted in the SEAT LCA studies. SEAT Deliverable Ref: D 3.5.* Annex report. Stirling, United Kingdom, SEAT Project. 121 pp. (available at http://media.leidenuniv.nl/legacy/d35-annexreport.pdf). (2014)

[26] Hornborg, S. & Ziegler, F. *Aquaculture and energy use: a desk-top study.* Gothenburg, University of Gothenburg. 19 pp. https://swemarc.gu.se/digitalAssets/1536/1536133_publication---energy-use-in-aquaculture.pdf (2014)

[27] Paterson, B. & Miller, S. Examining energy use in shrimp farming. *Global Aquaculture Advocate*, November 2014: 30–32. (2014)

[28] BEIS (Department for Business Energy & Industrial Strategy). *Government GHG conversion factors for company reporting: Methodology paper for emission factors.* London, UK Department of Business, Energy & Industrial Strategy. 112 pp. (available at https://assets.publishing.service.gov.uk/government/uploads/system/uploads/attachment_data/file/553488/2016_methodology_paper_Final_V01-00.pdf). (2016)
